# Polycystic ovary syndrome and extremely preterm birth: A nationwide register-based study

**DOI:** 10.1371/journal.pone.0246743

**Published:** 2021-02-04

**Authors:** Heiddis Valgeirsdottir, Inger Sundström Poromaa, Theodora Kunovac Kallak, Eszter Vanky, Tansim Akhter, Nathalie Roos, Olof Stephansson, Anna-Karin Wikström

**Affiliations:** 1 Department of Women’s and Children’s Health, Uppsala University, Uppsala, Sweden; 2 Faculty of Medicine and Health Sciences, Department of Clinical and Molecular Medicine, Norwegian University of Science and Technology, Trondheim, Norway; 3 Department of Obstetrics and Gynecology, St. Olav’s Hospital, Trondheim University Hospital, Trondheim, Norway; 4 Department of Medicine, Solna, Clinical Epidemiology Division, Karolinska Institute, Stockholm, Sweden; 5 Department of Women’s Health, Karolinska University Hospital, Stockholm, Sweden; University of Mississippi Medical Center, UNITED STATES

## Abstract

**Introduction:**

Women with polycystic ovary syndrome (PCOS) have increased risk of pregnancy complications, including preterm birth before 37 weeks. However, if this increased risk also includes extremely preterm births (<28 weeks) is unknown. Such information is important to identify women at risk and tailor antenatal care, since child morbidity and mortality become more prevalent with increasing prematurity.

**Aims:**

To investigate the association between PCOS and extremely preterm birth, and whether onset of PCOS-related preterm birth is predominantly spontaneous or medically indicated.

**Material and methods:**

This was a nationwide register-based cohort study in Sweden. The study population was all live singleton births registered in the Swedish Medical Birth Register 2005–2014 (n = 1 046 448). Women with and without PCOS were compared by severity of preterm birth [extremely (22+0 to 27+6 weeks), very (28+0 to 31+6 weeks) and moderately (32+0 to 36+6 weeks)] and delivery onset mode (spontaneous or medically indicated). Multinomial logistic regression was performed to estimate adjusted odds ratios (aOR) with 95% confidence intervals (CI). Adjustments were made for maternal age, parity, body mass index, smoking, country of birth and year of delivery.

**Results:**

During the study period, 1.3% of the women giving birth had PCOS diagnosis. They had an overall higher preterm birth rate than women without PCOS (6.7% and 4.8%, respectively). Women with PCOS had increased odds of preterm birth of all severities, with the highest odds for extremely preterm birth (aOR 2.3; 95% CI 1.7–3.0), particularly of spontaneous onset (aOR 2.7; 95% CI 2.0–3.6).

**Conclusions:**

Women with PCOS had more than a two-fold increased risk of extremely preterm birth with spontaneous onset than women without such diagnosis. This can be important in antenatal risk assessment of preterm birth in women with PCOS. Future research is warranted to investigate the biological mechanisms behind preterm birth in women with PCOS.

## Introduction

Polycystic ovary syndrome (PCOS) is an endocrine condition affecting 6–10% of women of reproductive age [1], and is characterized by menstrual irregularity, hyperandrogenism and polycystic ovaries [2]. Women with PCOS frequently present with central adiposity and insulin resistance, and are at increased risk of developing type 2 diabetes mellitus [3].

Preterm birth affects approximately 5–15% of all infants globally and is associated with substantial morbidity and mortality [4]. With decreasing gestational age, the risk of adverse health outcomes with long-term consequences for the infant, increases [5]. The etiology of preterm birth is complex and partly unknown [6]. Preterm birth can either have a spontaneous onset or be medically indicated due to maternal or fetal conditions, and the underlying mechanisms for these entities differ. Factors involved in the etiology of spontaneous preterm birth are inflammation, infection and uterine over-distension [7,8]. Hypertensive disorders, diabetes and fetal conditions, such as severe intrauterine growth restriction, are among the conditions behind medically indicated preterm birth.

Previous studies have reported that women with PCOS are at increased risk of preterm birth [9–12], but a number of pertinent questions remain unanswered. Previous studies have indicated that the risk increase of very preterm birth (<32 weeks) might be more pronounced than that of moderately preterm birth (32–36 weeks) [12,13], but information on extremely preterm birth is, to our knowledge, lacking. We also lack knowledge of whether the onset of PCOS-related preterm births is predominantly spontaneous or medically indicated. Only one previous study has investigated spontaneous and medically indicated preterm birth, suggesting that the rate of spontaneous-onset preterm delivery in women with PCOS was similar to that in women without PCOS [13]. Although extremely preterm birth is rare (0.7% of all births in the United States), it imposes a major burden to the individual and the family, as well as major financial costs for society [14].

The primary aim of this study was to investigate the association between PCOS and preterm birth by severity of prematurity, particularly extremely preterm birth, in a large population-based cohort including more than one million live births. A secondary aim was to study the association between PCOS and preterm birth according to onset of delivery.

## Materials and methods

The ethics committee did not waive the requirement for informed consent since the study is register-based and includes neither any deviation from clinical routine nor a direct contact with the study participants.

Prenatal care in Sweden is standardized and free of charge, and attendance is close to 100% [15]. During the first prenatal visit in the first trimester, the pregnant woman is interviewed about her medical, reproductive and obstetric history, as well as about smoking habits and civil status. Furthermore, maternal height and weight are measured.

The Swedish Medical Birth Register prospectively collects information on pregnant women as well as on pregnancy, delivery and the neonatal period, starting at the first prenatal visit. The register includes information on about 98% of all births in Sweden since 1973 [16] and information on pregnancy length is validated and considered fairly reliable [17]. Complications during pregnancy and delivery are classified, according to the International Classification of Diseases (ICD), by an obstetrician at the time of discharge from the delivery hospital. Information on each pregnancy and delivery is extracted from the standardized digital prenatal, obstetric and pediatric records, and forwarded to the Swedish Medical Birth Register. The Swedish Patient Register was established in 1964 (nationwide coverage since 1987) and includes information on dates of hospital admission, discharges and diagnoses classified according to ICD codes. Since 2001, this register also includes information on outpatient specialist care in both publicly and privately run settings [18]. The Total Population Register and the Swedish Education Register are held by Statistics Sweden and contribute with data on maternal country of birth and duration of formal education, respectively. The unique personal identification number assigned to each Swedish resident at birth or immigration [19], was used to link data from the Swedish Medical Birth Register to the other registers. The Swedish Board of Health and welfare undertake the linkage and the dataset received by the researchers is pesudonymized using a unique serial number.

### Study population

**Fig 1:**
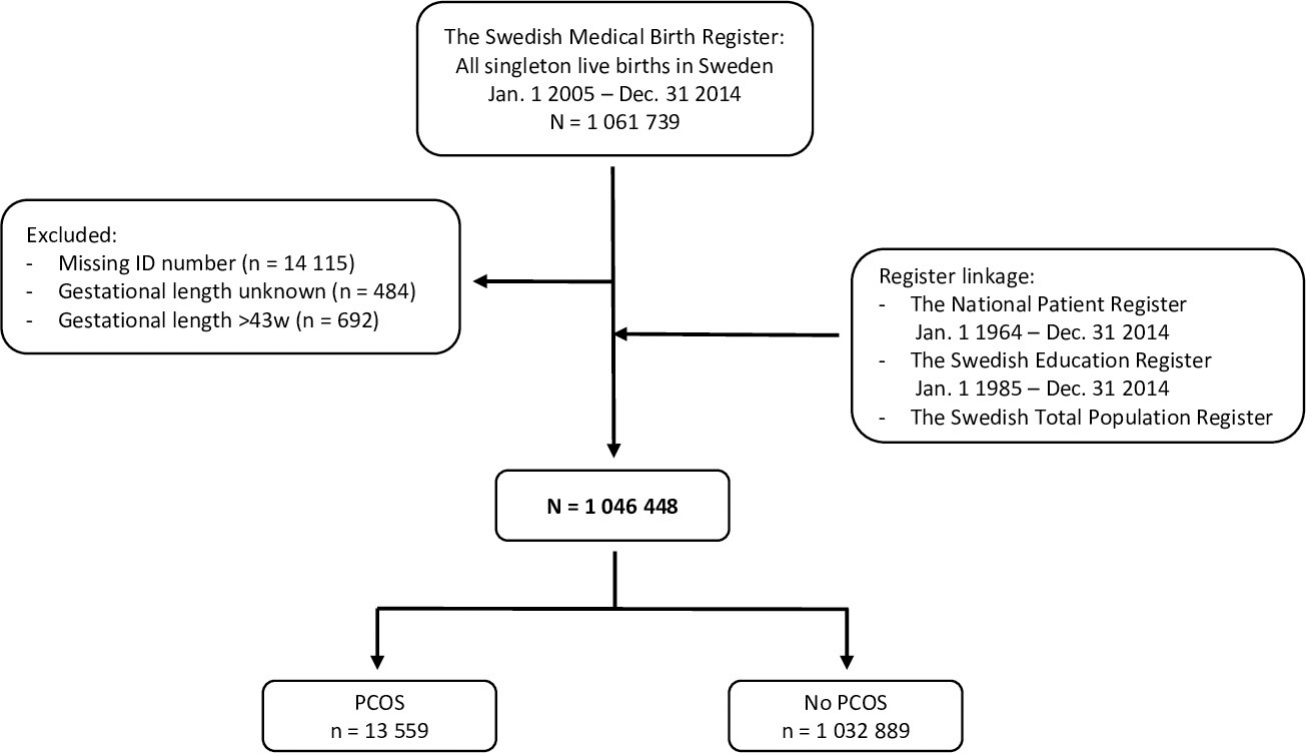
Flowchart of the study population.

All women with live born singleton pregnancies giving birth, to an infant at 22+0 gestational weeks or later, between 2005 and 2014 (n = 1 061 739) in Sweden were included in the study. The cohort was obtained from the Swedish Medical Birth Register. Pregnancies with unknown gestational age at birth (n = 484) or without personal identification number (n = 14 115) were excluded. According to clinical routines in Sweden during the study period, pregnancies should be induced before 43 weeks and 0 days. Based on this, women with gestational age more than 43 weeks and 6 days (n = 692) were excluded, assuming that the gestational age had been incorrectly registered. Thus, a total of 1 046 448 women were included in the final study cohort (Fig 1).

### Exposure

The exposure was diagnosis of PCOS identified in the Swedish Patient Register, until the date of discharge after the index delivery. PCOS diagnosis was identified according to the corresponding ICD codes (S1 Table). In 1990, the US National Institutes of Health (NIH) criteria for PCOS were introduced [20], and beginning in 2003, the Rotterdam criteria were swiftly introduced into clinical practice in Sweden [2], to replace the NIH criteria. The study period started in 2005, since it can be assumed that the Rotterdam criteria were generally established in medical practice in Sweden from that time point.

### Outcome

The primary outcome, preterm birth, was defined as gestational age <37 weeks at birth and was categorized, in accordance with the World Health Organization´s categorization, as extremely preterm birth (22+0 to 27+6 weeks), very preterm birth (28+0 to 31+6 weeks) or moderately preterm birth (32+0 and 36+6 weeks). The secondary outcome was preterm birth according to onset of delivery, categorized as spontaneous or medically indicated preterm birth. The categorization of onset of delivery was regardless of whether the child was subsequently delivered vaginally or with cesarean section. Information on onset of delivery is routinely recorded in a standardized manner (checkbox) by the delivery ward midwife and is categorized as spontaneous, induced vaginal or cesarean section before onset of labor. According to national guidelines [21], the definition of spontaneous onset of labor is when at least two of three following criteria are fulfilled: spontaneous regular contractions, rupture of membranes or cervix with full effacement and dilated more than 3 cm. In our study, we defined spontaneous onset of labor as all births recorded with spontaneous start (by checkbox) or if an ICD-diagnosis of preterm premature rupture of the membranes (PPROM) was present in the maternal records. Medically indicated onset was defined as those deliveries recorded as induced vaginal or had a cesarean section before onset of labor, unless a PPROM diagnosis was present. Information on mode of onset of delivery was missing in 4 409 women, 67 with and 4 342 without PCOS, respectively.

In Sweden, all pregnant women are offered a first- or early second-trimester ultrasound scan free of charge to accurately date the pregnancy and to scan for congenital anomalies. In this cohort, gestational age was determined according to the following hierarchy: first-trimester or early second-trimester scan (950 571 [90.8%]), date of last menstrual period reported at the first prenatal visit (46 365 [4.4%]) and postnatal assessment (49 512 [4.7%]).

### Covariates

Covariates were maternal age, parity, infertility treatment in index pregnancy, height, weight in early pregnancy, daily smoking in early pregnancy, cohabitation, reproductive history, presence of hypertensive or diabetic disease, country of birth, educational length and year of delivery. Early pregnancy maternal body mass index (BMI) was calculated, using the equation *weight* (*kg*)/*height* (*m*)^2^. Covariates were categorized according to Table 1. Hypertensive diseases were categorized as no hypertensive disease, chronic hypertension or pregnancy-induced hypertension (includes gestational hypertension, preeclampsia and eclampsia). Diabetic diseases were categorized as no diabetic disease, pregestational diabetes (diabetes type 1 and 2) and gestational diabetes. Screening for gestational diabetes in Sweden during the study period, was based on risk factors, except in one county (Region Skåne) that used universal oral glucose tolerance test. Diagnostic criteria for gestational diabetes varied between counties in Sweden, but ranged between glucose level 9.0–11.1 mmol/L, using either capillary or venous samples, obtained two hours after intake of 75 gram glucose solution, or a fasting glucose level between 6.1–7.0 mmol/L. During the study years, metformin was rarely used during pregnancy (for neither PCOS nor gestational diabetes). See S1 Table for ICD codes for hypertensive and diabetic diseases.

**Table 1:** Maternal characteristics in PCOS women giving birth in Sweden 2005–2014.

		PCOS			
		No		Yes	
	No. of women	n	(%)	n	(%)
**Total cohort**	1046448	1032889	(100)	13559	(100)
**Gestational length (weeks)**					
≥37	996383	983729	(95.2)	12654	(93.3)
32–36	43106	42375	(4.1)	731	(5.4)
28–31	4532	4439	(0.4)	93	(0.7)
22–27	2427	2346	(0.2)	81	(0.6)
**Age (years)**					
*Mean ± standard deviation*	*30*.*3 ± 5*.*2*	*30*.*3 ± 5*.*2*		*30*.*8 ± 4*.*7*	
12–24.9	151038	149798	(14.5)	1240	(9.1)
25–29.9	305210	301022	(29.1)	4188	(30.9)
30–34.9	363652	358542	(34.7)	5110	(37.7)
≥35	226540	223519	(21.6)	3021	(22.3)
Missing	8	8		0	
**Parity**					
1	464387	457663	(44.3)	6724	(49.6)
≥2	582051	575216	(55.7)	6835	(50.4)
Missing	10	10		0	
**Height (cm)**					
*Mean ± standard deviation*	*166*.*3 ± 6*.*5*	*166*.*3 ± 6*.*5*		*166*.*0 ± 6*.*5*	
<164	331350	326777	(33.2)	4573	(33.3)
164–171	453002	447223	(45.5)	5779	(45.5)
≥172	211936	209280	(21.3)	2656	(21.3)
Missing	50160	49609		551	
**BMI in early pregnancy (kg/m**^**2**^**)**					
*Mean ± standard deviation*	*24*.*7 ± 4*.*6*	*24*.*6 ± 4*.*6*		*26*.*9 ± 5*.*7*	
10.0–18.4	23076	22916	(2.4)	160	(1.3)
18.5–24.9	578606	573150	(60.2)	5456	(43.2)
25.0–29.9	244990	241484	(25.4)	3506	(27.7)
≥30	118526	115007	(12.1)	3519	(27.8)
Missing	81250	80379		918	
**Daily cigarette smoking in early pregnancy**					
No	931553	919296	(93.4)	12257	(94.2)
Yes	65770	65020	(6.6)	750	(5.8)
Missing	49125	48573		552	
**Cohabitation**					
Yes	936514	924168	(93.8)	12346	(95.1)
No	61625	60991	(6.2)	634	(4.9)
Missing	48309	47730		579	
**Involuntary childlessness before index pregnancy (years)**					
<1	955702	946844	(91.7)	8858	(65.3)
1–2	60546	57885	(5.6)	2661	(19.6)
≥3	30200	28160	(2.7)	2040	(15.0)
**Ovulation stimulation**					
No	1033731	1021936	(98.9)	11795	(87.0)
Yes	12717	10953	(1.1)	1764	(13.0)
**Other assisted reproduction treatment**					
No	1015685	1003968	(97.2)	11717	(86.4)
Yes	30763	28921	(2.8)	1842	(13.6)
**Hypertensive disease**					
No	1001905	989304	(95.8)	12601	(92.9)
Yes	4495	4340	(0.4)	155	(1.1)
Pregnancy induced hypertension^a^	40048	39245	(3.8)	803	(5.9)
**Diabetic disease**					
No	1029056	1016140	(98.4)	12916	(95.3)
Pregestational diabetes^b^	5991	5767	(0.6)	224	(1.7)
Gestational diabetes	11401	10982	(1.1)	419	(3.1)
**Country of birth**					
Sweden	805025	794781	(77.5)	10244	(76.1)
Other Nordic Country	15457	15250	(1.5)	207	(1.5)
Non-Nordic Country	218077	215065	(21.5)	3012	(22.4)
Missing	7889	7793		96	
**Education (years)**					
≤11	193276	190850	(18.6)	2426	(18.0)
12–15	420236	414070	(40.5)	5366	(39.7)
≥16	423825	418112	(40.8)	5713	(42.3)
Missing	9111	9057		54	
**Year of delivery in index pregnancy**					
2005–2008	400652	397383	(38.5)	3269	(24.1)
2009–2011	320840	316669	(30.7)	4171	(30.8)
2012–2014	324956	318837	(30.9)	6119	(45.1)

^a^Pregnancy-induced hypertension, preeclampsia or eclampsia.

^b^Type 1 or 2 diabetes.

### Statistical analysis

Logistic regression analysis was used to estimate the association between maternal PCOS and risk of preterm birth. In all analyses, crude and adjusted odds ratios (ORs) with 95% confidence intervals (CIs) were calculated using the generalized estimation equation method, as observations are not independent in women who gave birth more than once during the study period. In order to obtain systematic representation of causal relationships between PCOS diagnosis and preterm birth, as well as to decide which covariates should be included, we drew a directed acyclic graph (DAG, http://www.dagitty.net/) [22] (S1 Fig). In the first adjusted analysis (adjusted model 1), adjustment was made for covariates that were ancestors both of the exposure and the outcome according to the DAG, i.e. maternal age, country of birth and year of delivery. Adjustment was also made for parity and smoking habits since these were found to be confounders in our cohort, as shown in Tables 1 and 2. In the second adjusted analysis (adjusted model 2), we adjusted for the same covariates as in adjusted model 1 and additionally for BMI, since BMI can be regarded both as a confounder and a mediator. All covariates were included in the statistical models as categorical variables according to Table 1. Cases with missing data on covariates were excluded in the multivariable analyses.

**Table 2:** Maternal characteristics and rates of preterm birth in women giving birth in Sweden 2005–2014.

		Gestational age at delivery (weeks)							
		22–27		28–31		32–36		≥ 37	
	No. of women	n	(%)	n	(%)	n	(%)	n	(%)
**Total cohort**	1046448	2427	(100)	4532	(100)	43106	(100)	996383	(100)
**PCOS**									
No	1032889	2346	(96.7)	4439	(97.9)	42375	(98.3)	983729	(98.7)
Yes	13559	81	(3.3)	93	(2.1)	731	(1.7)	12654	(1.3)
**Age (years)**									
*Mean ± standard deviation*	*30*.*3 ± 5*.*2*	*30*.*5 ± 6*.*0*		*30*.*6 ± 5*.*7*		*30*.*2 ± 5*.*5*		*30*.*3 ± 5*.*2*	
12–24.9	151038	436	(18.0)	693	(15.3)	6962	(16.2)	142947	(14.3)
25–29.9	305210	621	(25.6)	1213	(26.8)	12564	(29.1)	290812	(29.2)
30–34.9	363652	732	(30.2)	1463	(32.3)	13759	(31.9)	347698	(34.9)
≥35	226540	638	(26.3)	1163	(25.7)	9820	(22.8)	214919	(21.6)
Missing	8	0		0		1		7	
**Parity**									
1	464387	1389	(57.2)	2558	(56.4)	23107	(53.6)	437333	(43.9)
≥2	582051	1038	(42.8)	1974	(43.6)	19998	(46.4)	559041	(56.1)
Missing	10	0		0		1		9	
**Height (cm)**									
*Mean ± standard deviation*	*166*.*3 ± 6*.*5*	*165*.*3 ± 6*.*8*		*165*.*2 ± 6*.*6*		*165*.*3 ± 6*.*6*		*166*.*3 ± 6*.*5*	
≤163	331350	725	(39.5)	1531	(38.4)	15752	(39.4)	313342	(33.0)
164–171	453002	777	(42.4)	1795	(45.0)	17325	(43.4)	433105	(45.6)
≥172	211936	332	(18.1)	662	(16.6)	6881	(17.2)	204061	(21.5)
Missing	50160	593		544		3148		45875	
**BMI in early pregnancy (kg/m**^**2**^**)**									
*Mean ± standard deviation*	*24*.*7 ± 4*.*6*	*25*.*8 ± 5*.*6*		*25*.*5 ± 5*.*4*		*24*.*9 ± 5*.*0*		*24*.*6 ± 4*.*6*	
10.0–18.4	23076	45	(2.5)	111	(2.9)	1278	(3.3)	21642	(2.4)
18.5–24.9	578606	917	(51.5)	2013	(52.2)	21868	(56.6)	553808	(60.1)
25.0–29.9	244990	470	(26.4)	1041	(27.0)	9850	(25.5)	233629	(25.4)
≥30	118526	348	(19.6)	695	(18.0)	5659	(14.6)	111824	(12.1)
Missing	81250	647		672		4451		75480	
**Daily cigarette smoking in early pregnancy**									
No	931553	1630	(88.8)	3579	(89.5)	36379	(91.0)	889965	(93.5)
Yes	65770	205	(11.2)	419	(10.5)	3617	(9.0)	61529	(6.5)
Missing	49125	592		534		3110		44889	
**Cohabitation**									
Yes	936514	1679	(91.7)	3632	(90.9)	37057	(92.5)	894146	(93.3)
No	61625	152	(8.3)	362	(9.1)	3021	(7.5)	58090	(6.1)
Missing	48309	596		538		3028		44147	
**Involuntary childlessness before index pregnancy (years)**									
<1	955702	2143	(88.3)	3910	(86.3)	38256	(88.7)	911393	(91.5)
1–2	60546	149	(6.1)	350	(7.7)	3075	(7.1)	56972	(5.7)
≥3	30200	135	(5.6)	272	(6.0)	1775	(4.1)	28018	(2.8)
**Ovulation stimulation**									
No	1033731	2389	(98.4)	4439	(97.9)	42459	(98.5)	984444	(98.8)
Yes	12717	38	(1.6)	93	(2.1)	647	(1.5)	11939	(1.2)
**Other assisted reproduction treatment**									
No	1015685	2304	(94.9)	4307	(95.0)	41336	(95.9)	967738	(97.1)
Yes	30763	123	(5.1)	225	(5.0)	1770	(4.1)	28645	(2.9)
**Hypertensive disease**									
No	1001905	2012	(82.9)	3260	(71.9)	37297	(86.5)	959336	(96.3)
Chronic hypertension	4495	74	(3.0)	126	(2.8)	509	(1.2)	3786	(0.4)
Pregnancy induced hypertension^a^	40048	341	(14.1)	1146	(25.3)	5300	(12.3)	33261	(3.3)
**Diabetic disease**									
No	1029056	2383	(98.2)	4360	(96.2)	41026	(95.2)	981287	(98.5)
Pregestational diabetes^b^	5991	21	(0.9)	83	(1.8)	1150	(2.7)	4737	(0.5)
Gestational diabetes	11401	23	(0.9)	89	(2.0)	930	(2.2)	10359	(1.0)
**Country of birth**									
Sweden	805025	1694	(71.2)	3433	(76.5)	33446	(78.2)	766452	(77.5)
Other Nordic Country	15457	39	(1.6)	63	(1.4)	588	(1.4)	14767	(1.5)
Non-Nordic Country	218077	646	(27.2)	991	(22.1)	8749	(20.4)	207691	(21.1)
Missing	7889	48		45		323		7473	
**Education (years)**									
≤11	193276	619	(25.8)	1098	(24.5)	9156	(21.4)	182403	(18.5)
12–15	420236	1027	(42.8)	1846	(41.1)	17771	(41.6)	399592	(40.5)
≥16	423825	753	(31.8)	1544	(34.4)	15832	(37.0)	405696	(41.1)
Missing	9111	28		44		347		8692	
**Year of delivery in index pregnancy**									
2005–2008	400652	915	(37.7)	1850	(40.8)	16956	(39.3)	380931	(38.2)
2009–2011	320840	767	(31.6)	1349	(29.8)	13263	(30.8)	305461	(30.7)
2012–2014	324956	745	(30.7)	1333	(29.4)	12887	(29.9)	309991	(31.1)

^a^Pregnancy-induced hypertension, preeclampsia or eclampsia.

^b^Type 1 or 2 diabetes.

As a sensitivity analysis, we calculated the risk of preterm birth in PCOS women, where we even adjusted for infertility treatment (ovulation stimulation and other assisted reproduction treatment) in the adjusted model 2.

We also wanted to investigate whether the preterm birth risk in PCOS women was mainly explained by PCOS-related disorders such as hypertension and diabetes. We therefore calculated the risk of preterm birth in PCOS women after excluding women with hypertensive and diabetic diseases (both pre-gestational and gestational disorders).

Finally, we restricted our population to primiparous women and estimated the association between PCOS and risk for preterm birth by severity.

When analyzing the risk of very preterm birth, women who had given birth extremely preterm were excluded since they were no longer at risk of giving birth. Similarly, when analyzing the risk of moderately preterm birth, women who had given birth before 32 weeks of gestation were excluded.

Data were analyzed using IBM SPSS 25 and SAS software version 9.4. P-values were two-sided and p-values <0.05 were considered statistically significant.

The study was approved by the Regional Ethics Committee at the Karolinska Institute, Stockholm, Sweden (2008/1182-31/4 and 2011/1856-32). All data were fully pseudonymized before our access to them.

## Results

### Maternal characteristics and PCOS

Among all women giving birth to singletons in Sweden during 2005–2014, 13 559 (1.3%) had a PCOS diagnosis. PCOS diagnosis became more frequent as the years passed; 0.8% of the women giving birth in Sweden during 2005–2008 had a PCOS diagnosis, while the corresponding proportion was 1.9% during 2012–2014. Maternal characteristics in women with and without PCOS diagnosis are shown in Table 1. Women with PCOS were slightly older, more often primiparous, had higher BMI, smoked less frequently and were more often cohabiting than women without PCOS. Furthermore, more than one year of involuntary childlessness before the index pregnancy (infertility), as well as conception after ovulation stimulation or other assisted reproduction treatment were more common in women with PCOS than in those without. Comorbidity, i.e. hypertensive and diabetic disease, were also more common in women with PCOS. It was slightly more common that women with PCOS were born in non-Nordic countries and they generally had a higher level of education.

### Preterm birth

In the final cohort of 1 046 448 women, 4.8% delivered preterm (<37 weeks); 4.1% moderately preterm (32w0d to 36w6d), 0.4% very preterm (28w0d to 31w6d) and 0.2% extremely preterm (22w0d to 27w6d). Preterm birth was more common in women with PCOS (6.7%, n = 905 of 13 559 women) than in women without PCOS (4.8%, n = 49 160 of 1 032 889 women). Of 13 559 births in women with PCOS, 81 (0.6%) were extremely preterm, 93 (0.7%) very preterm and 731 (5.4%) moderately preterm. The corresponding proportions were 0.2%, 0.4% and 4.1%, respectively, in women without PCOS.

Comparisons of maternal characteristics according to preterm severity are shown in Table 2. Women who delivered preterm had PCOS more frequently, were more often either in the youngest or the oldest age categories, were more often primiparous, their mean height was lower, they had higher mean BMI and were more often smokers. They also more often had hypertensive or diabetic disease, both pre-gestational and pregnancy-induced disorders.

Maternal characteristics in women with PCOS giving birth preterm compared to term are shown in S2 Table. Women with PCOS who delivered preterm were more often primipara, had higher mean BMI, more often smoked, had higher rates of infertility treatment and were more often born in countries outside of the Nordic countries than women with PCOS and term birth.

Maternal PCOS was associated with a 43% higher risk of preterm birth (crude OR: 1.43, 95% CI 1.34–1.53). The association remained at the same level when we adjusted for maternal age, parity, smoking habits, country of birth and year of delivery (adjusted OR [model 1] = 1.41, 95% CI 1.31–1.52) and marginally decreased when we added BMI into the model (adjusted OR [model 2] = 1.34, 95% CI 1.24–1.45).

In a sensitivity analysis, with adjustment for even infertility treatment (ovulation stimulation and other assisted reproduction treatment) in model 2, the association was slightly weaker (adjusted OR 1.27, 95% CI 1.18–1.37).

Table 3 illustrates the associations between maternal PCOS and preterm severity. The association was stronger with increasing severity of preterm birth, with an adjusted OR (model 1) for extremely preterm birth (<28 weeks) of 2.65 (95% CI 2.04–3.44). The risk was slightly attenuated after also adjusting for BMI (model 2).

**Table 3:** Risk of preterm birth, according to severity and PCOS diagnosis, in women giving birth in Sweden 2005–2014.

		Odds ratio (95% Confidence Interval)			
	n (%)	Crude	Model 1^a^	Model 2^b^	Restricted^c^
**Extremely preterm birth (22–27 weeks)**					
no PCOS	2346 (0.23)	1.00	1.00	1.00	1.00
PCOS	81 (0.60)	2.64 (2.11–3.30)	2.65 (2.04–3.44)	2.28 (1.74–2.99)	2.42 (1.80–3.25)
**Very preterm birth (28–31 weeks)**					
no PCOS	4439 (0.43)	1.00	1.00	1.00	1.00
PCOS	93 (0.69)	1.61 (1.30–1.98)	1.55 (1.24–1.95)	1.42 (1.13–1.78)	1.67 (1.27–2.19)
**Moderately preterm birth (32–36 weeks)**					
no PCOS	42375 (4.13)	1.00	1.00	1.00	1.00
PCOS	731 (5.46)	1.34 (1.24–1.45)	1.33 (1.23–1.45)	1.28 (1.18–1.39)	1.28 (1.17–1.40)

^a^Adjusted for maternal age, parity, smoking habits, country of birth and year of delivery.

^b^Adjusted for same covariates as model 1, plus for BMI.

^c^Adjusted for same covariates as model 2 and women with hypertensive or diabetic diseases are excluded.

When the population was restricted to primiparous women, we found a similar association between PCOS and extremely preterm birth (adjusted OR [model 1] = 3.13, 95% CI 2.31–4.24 and adjusted OR [model 2] = 2.66, 95% CI 1.95–3.64) as in the whole population (Table 3). Presented in S2 Table.

### Mode of onset of delivery

Among the 13 495 women with PCOS and information on onset of delivery, 623 (4.6%) had spontaneous preterm birth and 274 (2.0%) had medically indicated preterm birth. The corresponding numbers in women without PCOS (n = 1 028 547) were 34 649 (3.4%) for spontaneous preterm birth and 13 896 (1.4%) for medically indicated preterm birth. Of all the preterm deliveries (<37 weeks) with spontaneous onset (n = 35 272), 14 590 (41.4%) started with spontaneous rupture of membranes (PPROM).

Maternal PCOS was associated with increased risk of both spontaneous and medically indicated preterm birth (adjusted OR [model 2] = 1.36, 95% CI 1.24–1.48 and adjusted OR [model 2] = 1.31, 95% CI 1.15–1.50, respectively).

Table 4 illustrates the association between maternal PCOS and preterm birth severity, according to onset of delivery. For spontaneous preterm birth, PCOS was associated with all preterm severities, but the association was strongest for extremely preterm birth (adjusted OR [model 1] = 3.06, 95% CI 2.27–4.11). Adjusting also for BMI (model 2) marginally decreased the association (adjusted OR [model 2] = 2.66, 95% CI 1.95–3.62). The association between PCOS and medically indicated preterm birth was statistically significant for moderately preterm birth (adjusted OR [model 1] = 1.50, 95% CI 1.30–1.73) and only marginally significant for extremely preterm birth (adjusted OR [model 1] = 1.78 (1.01–3.16). The association with moderately preterm birth was slightly attenuated after also adjusting for BMI (model 2) and was no longer significant for extremely preterm birth.

**Table 4:** Risk of preterm birth in women with PCOS by mode of onset of delivery; preterm birth divided into extremely, very and moderately preterm.

		Odds ratio (95% Confidence Interval)			
	n (%)	Crude	Model 1^a^	Model 2^b^	Restricted^c^
**Spontaneous extremely preterm birth (22–27 weeks)**					
no PCOS	1585 (0.15)	1.00	1.00	1.00	1.00
PCOS	61 (0.45)	2.94 (2.26–3.84)	3.06 (2.27–4.11)	2.66 (1.95–3.62)	2.53 (1.82–3.52)
**Spontaneous very preterm birth (28–31 weeks)**					
no PCOS	2362 (0.23)	1.00	1.00	1.00	1.00
PCOS	60 (0.44)	1.95 (1.51–2.52)	1.87 (1.42–2.48)	1.86 (1.40–2.47)	1.86 (1.38–2.50)
**Spontaneous moderately preterm birth (32–36 weeks)**					
no PCOS	30702 (2.98)	1.00	1.00	1.00	1.00
PCOS	502 (3.72)	1.27 (1.16–1.40)	1.27 (1.16–1.39)	1.26 (1.14–1.38)	1.25 (1.13–1.38)
**Medically indicated extremely preterm birth (22–27 weeks)**					
no PCOS	669 (0.07)	1.00	1.00	1.00	1.00
PCOS	19 (0.14)	2.17 (1.38–3.43)	1.78 (1.01–3.16)	1.55 (0.87–2.75)	1.96 (0.88–4.39)
**Medically indicated very preterm birth (28–31 weeks)**					
no PCOS	1980 (0.19)	1.00	1.00	1.00	1.00
PCOS	30 (0.22)	1.16 (0.81–1.67)	1.14 (0.78–1.68)	0.96 (0.65–1.42)	0.97 (0.48–1.95)
**Medically indicated moderately preterm birth (32–36 weeks)**					
no PCOS	11247 (1.09)	1.00	1.00	1.00	1.00
PCOS	225 (1.67)	1.54 (1.35–1.76)	1.50 (1.30–1.73)	1.36 (1.17–1.57)	1.33 (1.07–1.64)

Information on mode of delivery was missing in 4409 cases, 67 with PCOS and 4342 without PCOS.

^a^Adjusted for maternal age, parity, smoking habits, country of birth and year of delivery.

^b^Adjusted for same covariates as model 1, plus for BMI.

^c^Adjusted for same covariates as model 2 and women with hypertensive or diabetic diseases are excluded.

For all analyses, excluding women with diabetic or hypertensive disease failed to affect any of the observed associations (see restricted analysis in Tables 3 and 4), and there was no observed effect on the difference between spontaneous onset and medically indicated onset of preterm delivery.

## Discussion

### Principal findings

In our nationwide cohort study of more than one million births, we found that PCOS was associated with increased risk of preterm birth. The highest risk-increase was observed for extremely preterm birth (<28 weeks), particularly with spontaneous onset. This finding is of clinical importance, since the risks of infant morbidity and mortality increase with decreasing gestational age [4,5].

### Strengths and limitations

The main strength of the study was the large cohort, in which all singleton pregnancies in Sweden during the study period were included and enabled further stratification of the cohort. Another strength is the prospective collection of data. Earlier studies on the risk of preterm birth in women with PCOS are heterogeneous in their design when it comes to both study populations and confounders. Some studies include only women with PCOS undergoing assisted reproductive treatment [23,24], thus lacking information about the total pregnant population with PCOS. A number of recent studies that have indicated associations between PCOS and preterm birth lack adjustments for possible cofounders such as smoking [13,23,24], which is an important risk factor for preterm birth, which we were able to include in our analyses. Clinical diagnoses, such as PCOS and hypertensive and diabetic disease, were determined by a medical professional, and hence more reliable than if self-reported.

An important limitation is the low registered prevalence of PCOS. This is related to the incomplete reporting of women with PCOS to the Patient Register. In order for women to obtain a PCOS diagnosis, they had to have sought specialized medical care for their symptoms or infertility and women with no or low degree of symptoms will not seek care. This results in a proportion of the non-PCOS group represents women with PCOS but not diagnosed. This entails that our findings might be more relevant for women with severe form of PCOS. We expect however that the misclassified group is small compared to the whole non-PCOS group, and that the effect therefore will be marginal. Another reason to the low prevalence of PCOS in our population might be a selection bias, with inclusion only of women who are fertile and have given birth. Since PCOS is associated with infertility, the prevalence will be underestimated. A further limitation is that no information on PCOS phenotype was available, as it is not captured by ICD coding.

### Comparison with earlier studies

A recent systematic review and meta-analysis [10], and a comprehensive review from 2015 [9], show increased risk of preterm birth in women with PCOS, compared to women without PCOS and our research confirms this association. There are prior reports suggesting that women with PCOS have an even higher risk of very preterm birth than of moderately preterm birth [12,13]. Our study expands this knowledge by demonstrating an association between PCOS and extremely preterm birth, a condition entailing high infant mortality, as well as both short- and long-term morbidity. Furthermore, we add to the knowledge that PCOS-related preterm birth, particularly of the most severe degree, are predominantly of spontaneous onset. Earlier studies indicating no significantly increased risk of preterm birth in women with PCOS were often based on limited study populations and did not differ between spontaneous and medically indicated onset of preterm birth [10,25].

Earlier studies on the risk of preterm birth in women with PCOS are heterogeneous in their design when it comes to both study populations and confounders. Some studies include only women undergoing assisted reproductive treatment [23,24], thus lacking information about the whole pregnant population with PCOS. A number of recent studies that have indicated associations between PCOS and preterm birth lack adjustments for possible cofounders such as smoking [13,23,24], which was, on the other hand, considered in our study.

### Interpretations

We found a positive association between PCOS and the more severe degrees of spontaneous preterm birth. Still, the mechanism causing preterm birth in women with PCOS is unknown. PCOS is associated with low-grade adipose-tissue-related systematic inflammation [26,27], and spontaneous preterm birth has also been associated with chronic inflammation [28]. Preterm birth has been associated with increased levels of pro-inflammatory proteins such as interleukin 1 [29], and 6 [30] and tumor necrosis factor [31]. Pro-inflammation in women with PCOS is demonstrated by elevated inflammatory markers such as C-reactive protein [32], interleukin-18, monocyte chemoattractant protein-1 [26], transforming growth factor-β1 and nuclear factor-κβ [27]. Palomba et al. have found that low-grade inflammatory state in PCOS women persists and increases during pregnancy [33]. Furthermore, metformin seems to have anti-inflammatory effects, even in non-diabetic individuals [34]. Treatment with metformin reduced the risk of both late miscarriage and preterm delivery in women with PCOS, and the beneficial effect in individual pooled data is seen in both hyper- and normoandrogenic women [11].

Overweight and obesity are known risk factors for preterm birth [35], and women with POCS tend to have higher BMI than women without PCOS [36]. When we adjusted for BMI in the association between PCOS and preterm birth, the association was only slightly attenuated. This finding suggests that the difference in BMI between PCOS- and non-PCOS women does not completely explain the association between PCOS and preterm birth. This trend is seen in the risk assessment for all preterm severities. We noticed a slightly more prominent effect when adjusted for BMI in the preterm births with medically indicated onset compared to spontaneous onset.

When we restricted our population from women with hypertensive and diabetic diseases, the association between PCOS and preterm birth was slightly weakened but remained significant. We can thus confirm that increased risk of medically indicated preterm birth is probably not caused by hypertensive and diabetic disease in women with PCOS. Further, when adjusting for even infertility treatment in the regression model, the estimate changed only marginally suggesting the association of PCOS and preterm birth is not fully explained by these factors either.

Hyperandrogenism might also be an important predictor of preterm birth in women with PCOS [24]. It has been hypothesized that androgens may influence remodeling and ripening of the cervix at term, and perhaps in preterm deliveries as well [37]. Future studies on the risk of preterm birth in women with different phenotypes of PCOS might add important information on the role of hyperandrogenism in preterm birth in women with PCOS.

## Conclusion

Women with PCOS are at more than a twofold increased risk of extremely preterm birth, compared to women without PCOS. The association was particularly strong for spontaneous onset of extremely preterm delivery, for which the risk was increased threefold. This can be important in antenatal risk assessment of preterm birth in women with PCOS. Future research is warranted to investigate the pathogenic mechanisms between PCOS and preterm birth.

## Supporting information

S1 Fig Directed Acyclic Graph (DAG) for systematic representation of causal relationships between PCOS diagnosis and preterm birth. (TIF)

S1 Table Classification of diseases during pregnancy according to the International Classification of Diseases (ICD). (DOCX)

S2 Table Maternal characteristics in women with PCOS giving birth preterm compared with term in Sweden during 2005–2014. (DOCX)

S3 Table Restricted analysis. Risk for severity of preterm birth in primiparous women giving birth in Sweden during 2005–2014 by PCOS diagnosis.
(DOCX)
