# Supplementary figures and images for: Polycystic ovary syndrome and extremely preterm birth: A nationwide register-based study
Source: PLoS One. 2021 Feb 4;16(2):e0246743. doi: 10.1371/journal.pone.0246743 (PMC7861420; doi:10.1371/journal.pone.0246743)

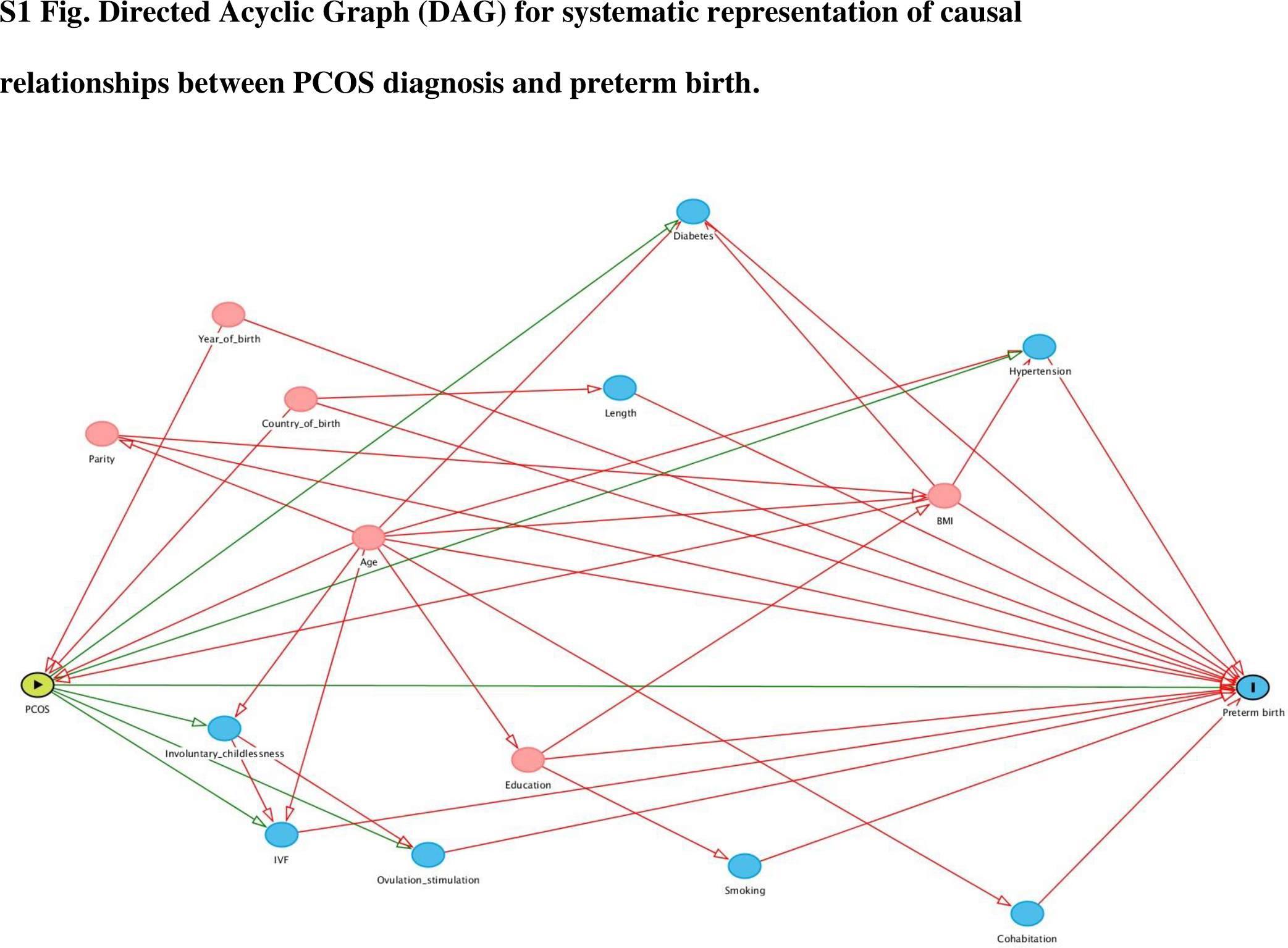

Supplement: S1 Fig — (TIF) [file pone.0246743.s001.tif]
