# Supplementary material for: Polycystic ovary syndrome and extremely preterm birth: A nationwide register-based study
Source: PLoS One. 2021 Feb 4;16(2):e0246743. doi: 10.1371/journal.pone.0246743 (PMC7861420; doi:10.1371/journal.pone.0246743)
Supplement: S1 Table — (DOCX) [file pone.0246743.s002.docx]

**Table S1**. **Classification of diseases during pregnancy according to the International Classification of Diseases (ICD)**.

8th revision for years 1973-1986; 9th revision for years 1987-1996; and 10th revision from 1997.

| **Diagnosis** | **ICD-8** | **ICD-9** | **ICD-10** |  |  |  |  |  |
| --- | --- | --- | --- | --- | --- | --- | --- | --- |
| PCOS | 256.90 | 256E | E28.2 |  |  |  |  |  |
| PPROM |  |  | O42 |  |  |  |  |  |
| Chronic hypertension |  |  | I10-15 |  |  |  |  |  |
|  |  |  | O10-11 |  |  |  |  |  |
| Gestational hypertension | |  | O13 |  |  |  |  |  |
| Preeclampsia |  |  | O11 |  |  |  |  |  |
|  |  |  | O14 |  |  |  |  |  |
| Eclampsia |  |  | O15 |  |  |  |  |  |
| Gestational diabets |  |  | O244 |  |  |  |  |  |
| Pregestational diabetes | |  | E10-13 |  |  |  |  |  |
|  |  |  | O240-243 |  |  |  |  |  |
