# Supplementary material for: Polycystic ovary syndrome and extremely preterm birth: A nationwide register-based study
Source: PLoS One. 2021 Feb 4;16(2):e0246743. doi: 10.1371/journal.pone.0246743 (PMC7861420; doi:10.1371/journal.pone.0246743)
Supplement: S2 Table — (DOCX) [file pone.0246743.s003.docx]

**S2 Table. Maternal characteristics in women with PCOS giving birth preterm compared with term in Sweden during 2005-2014.**

|  |  | **PCOS women with preterm delivery (weeks)** | | | **PCOS women with term delivery (weeks)** |
| --- | --- | --- | --- | --- | --- |
|  |  | **22-27** | **28-31** | **32-36** | **≥ 37** |
|  | **No. Of Women** | **n (%)** | **n (%)** | **n (%)** | **n (%)** |
| **Total cohort** | 13559 | 81 | 93 | 731 | 12654 |
| **Age (years)** | |  |  |  |  |
| *Mean ± SD* | *30.8 ± 4.7* | *30.9 ± 5.3* | *31.2 ± 4.5* | *30.6 ± 4.7* | *30.8 ± 4.7* |
| 10-24.9 | 1240 | 9 (11.1) | 7 (7.5) | 74 (10.1) | 1150 (9.1) |
| 25-29.9 | 4188 | 28 (34.6) | 29 (31.2) | 239 (32.7) | 3892 (30.8) |
| 30-34.9 | 5110 | 26 (32.1) | 35 (37.6) | 266 (36.4) | 4783 (37.8) |
| ≥35 | 3021 | 18 (22.2) | 22 (23.7) | 152 (20.8) | 2829 (22.4) |
| **Parity** | |  |  |  |  |
| 1 | 6724 | 61 (75.3) | 64 (68.8) | 413 (56.5) | 6186 (48.9) |
| ≥2 | 6835 | 20 (24.7) | 29 (31.2) | 318 (43.5) | 6468 (51.1) |
| **Height (cm)** | |  |  |  |  |
| *Mean ± SD* | *166.0 ± 6.5* | *163.8 ± 7.5* | *165.0 ± 6.0* | *164.8 ± 6.5* | *166.1 ± 6.5* |
| ≤163 | 4573 | 30 (47.6) | 31 (37.8) | 292 (42.3) | 4220 (34.7) |
| 164-171 | 5779 | 23 (36.5) | 40(48.8) | 298 (43.2) | 5418 (44.5) |
| ≥172 | 2656 | 10 (15.9) | 11 (13.4) | 100 (14.5) | 2535 (20.8) |
| Missing | 551 | 18 | 11 | 41 | 481 |
| **BMI in early pregnancy (kg/m^2^)** | |  |  |  |  |
| *Mean ± SD* | *26.9 ± 5.7* | *29.1 ± 6.0* | *28.3 ± 5.8* | *27.9 ± 6.2* | *26.8±5.7* |
| 10.0-18.4 | 160 | 1 (1.7) | 0 | 13 (1.9) | 146 (1.2) |
| 18.5-24.9 | 5456 | 14 (23.7) | 26 (32.5) | 245 (36.5) | 5171 (43.7) |
| 25.0-29.9 | 3506 | 23 (39.0) | 24 (30.0) | 175 (26.0) | 3284 (27.8) |
| ≥30 | 3519 | 21 (35.6) | 30 (37.5) | 239 (35.6) | 3229 (27.3) |
| Missing | 918 | 22 | 13 | 59 | 824 |
| **Daily cigarette smoking in early pregnancy** | |  |  |  |  |
| No | 12257 | 57 (90.5) | 76 (93.8) | 635 (92.4) | 11489 (94.4) |
| Yes | 750 | 6 (9.5) | 5 (6.2) | 687 (5.6) | 687 (5.6) |
| Missing | 552 | 18 | 12 | 44 | 478 |
| **Cohabitation** | |  |  |  |  |
| Yes | 12346 | 61 (96.8) | 74 (92.5) | 653 (94.0) | 11558 (95.2) |
| No | 634 | 2 (3.2) | 6 (7.5) | 42 (6.0) | 584 (4.8) |
| Missing | 579 | 18 | 13 | 36 | 512 |
| **Involuntary childlessness before index pregnancy (years)** | |  |  |  |  |
| <1 | 8858 | 44 (54.3) | 62 (66.7) | 442 (60.5) | 8310 (65.7) |
| 1-2 | 2261 | 12 (14.8) | 16 (17.2) | 154 (21.1) | 2479 (19.6) |
| ≥3 | 2040 | 25 (30.9) | 15 (16.1) | 135 (18.5) | 1865 (14.7) |
| **Ovulation stimulation** | |  |  |  |  |
| No | 11795 | 70 (86.4) | 83 (89.2) | 648 (88.6) | 10994 (86.9) |
| Yes | 1764 | 11 (13.6) | 10 (10.8) | 83 (11.4) | 1660 (13.1) |
| **Other assisted reproduction treatment** | |  |  |  |  |
| No | 11717 | 61 (75.3) | 80 (86.0) | 592 (81.0) | 10984 (86.8) |
| Yes | 1842 | 20 (24.7) | 13 (14.0) | 139 (19.0) | 1670 (13.2) |
| **Hypertensive disease** | |  |  |  |  |
| No | 12601 | 69 (85.2) | 67 (72.0) | 614 (84.0) | 11851 (93.7) |
| Chronic hypertension | 155 | 5 (6.2) | 4 (4.3) | 18 (2.5) | 128 (1.0) |
| Pregnancy induced hypertension^a^ | 803 | 7 (8.6) | 22 (23.7) | 99 (13.5) | 675 (5.3) |
| **Diabetic disease** | |  |  |  |  |
| No | 12916 | 78 (96.3) | 85 (91.4) | 650 (88.9) | 12103 (95.6) |
| Pregestational diabetes^b^ | 224 | 2 (2.5) | 7 (7.5) | 75 (5.7) | 173 (1.4) |
| Gestational diabetes | 419 | 1 (1.2) | 1 (1.1) | 39 (5.3) | 378 (3.0) |
| **Country of birth** | |  |  |  |  |
| Sweden | 10244 | 51 (65.4) | 63 (68.5) | 548 (75.6) | 9582 (75.2) |
| Other Nordic Country | 207 | 1 (1.3) | 3 (3.3) | 4 (0.6) | 199 (1.6) |
| Country outside of the Nordic Countries | 3012 | 26 (33.3) | 26 (28.3) | 173 (23.9) | 2787 (22.2) |
| Missing | 96 | 3 | 1 | 6 | 86 |
| **Education (years)** | |  |  |  |  |
| ≤11 | 2426 | 18 (22.5) | 25 (26.9) | 160 (21.9) | 2223 (17.6) |
| 12-15 | 5366 | 36 (45.0) | 33 (35.5) | 305 (41.8) | 4992 (39.6) |
| ≥16 | 5713 | 26 (32.5) | 35 (37.6) | 264 (36.2) | 5388 (42.8) |
| Missing | 54 | 1 | 0 | 2 | 51 |
| **Year of delivery in index pregnancy** | |  |  |  |  |
| 2005-2008 | 3269 | 27 (33.3) | 25 (26.9) | 198 (27.1) | 3019 (23.9) |
| 2009-2011 | 4171 | 25 (30.9) | 35 (37.6) | 221 (30.2) | 3890 (30.7) |
| 2012-2014 | 6119 | 29 (35.8) | 33 (35.5) | 312 (42.7) | 5745 (45.4) |
| **Onset of birth** | |  |  |  |  |
| Spontaneous | 9769 | 61 (76.3) | 60 (66.7) | 502 (69.1) | 9146 (72.6) |
| Medically indicated | 3723 | 19 (23.8) | 30 (33.3) | 225 (30.9) | 3449 (27.4) |
| Missing | 67 | 1 | 3 | 4 | 59 |

^a^Pregnancy induced hypertension, preeclampsia or eclampsia

^b^Type 1 or 2 diabetes
