# Supplementary material for: Polycystic ovary syndrome and extremely preterm birth: A nationwide register-based study
Source: PLoS One. 2021 Feb 4;16(2):e0246743. doi: 10.1371/journal.pone.0246743 (PMC7861420; doi:10.1371/journal.pone.0246743)
Supplement: S3 Table — Risk for severity of preterm birth in primiparous women giving birth in Sweden during 2005–2014 by PCOS diagnosis. (DOCX) [file pone.0246743.s004.docx]

**S3 Table. Restricted analysis. Risk for severity of preterm birth in primiparous women giving birth in Sweden during 2005-2014 by PCOS diagnosis.**

|  |  | **Odds ratio (95% Confidence Interval)** | |
| --- | --- | --- | --- |
|  | **n (%)** | **Model 1^a^** | **Model 2^b^** |
| **Extreme preterm birth (22-27 weeks)** | | |  |
| no PCOS | 1328 (0.29) | 1.00 | 1.00 |
| PCOS | 61 (0.91) | 3.13 (2.31-4.24) | 2.66 (1.95-3.64) |
| **Very preterm birth (28-31 weeks)** | | |  |
| no PCOS | 2494 (0.54) | 1.00 | 1.00 |
| PCOS | 64 (0.95) | 1.77 (1.36-2.31) | 1.58 (1.21-2.07) |
| **Moderate preterm birth (32-36 weeks)** | | |  |
| no PCOS | 22694 (4.96) | 1.00 | 1.00 |
| PCOS | 413 (6.14) | 1.29 (1.16-1.43) | 1.25 (1.13-1.39) |

^a^Adjusted for maternal age, smoking habits and country of birth and year of delivery

^b^Adjusted as Model 1, and even for BMI
